# Comparison of insulin requirements across gestation in women with hyperglycemia in pregnancy: A prospective cohort study

**DOI:** 10.3389/fendo.2022.1013663

**Published:** 2022-10-20

**Authors:** Chong Rao, Fan Ping

**Affiliations:** ^1^ Department of Endocrinology, Beijing ChuiYangLiu Hospital, Beijing, China; ^2^ Department of Endocrinology, Peking Union Medical College Hospital, Peking Union Medical College, Chinese Academy of Medical Sciences, Key Laboratory of Endocrinology Assigned by Ministry of Health, Beijing, China

**Keywords:** pre-gestational diabetes, gestation diabetes mellitus, preprandial insulin, total daily dose, pregnancy, postpartum

## Abstract

**Objectives:**

The aim of this study is to explore the daily insulin dose and the percentage change in preprandial and basal insulin dosage of women with different types of hyperglycemia in pregnancy (HIP) during the whole gestation and postpartum period.

**Methods:**

A total of 121 subjects with HIP requiring insulin therapy were enrolled from a prospective cohort consisted of 436 pregnant women with hyperglycemia. The subjects were divided into three groups: Group 1 [type 1 diabetes mellitus (T1DM) and maturity onset diabetes of the young (MODY)], Group 2 [type 1 diabetes mellitus (T2DM)], and Group 3 [gestation diabetes mellitus (GDM)]. The primary study measurements included daily dose and percentage of different types of exogenous insulin requirements across gestation in different groups.

**Results:**

Insulin total daily dosage of Group 1 was highest among the three groups and increased significantly from the first to the second/third trimester. Percentage of preprandial insulin increased from 53.8% (46.7, 60.0) and 54.5% (42.3, 62.9) in the first trimester to 63.6% (54.9, 75.0) and 67.2% (51.8, 73.7) in the second/third trimester in Group 1 and Group 2. All subjects with T1DM and 18.6% of subjects with T2DM still required insulin administration after delivery, with a 26.9% (19.0, 46.0) and 36.7% (26.9, 52.6) decrease in total insulin dose, respectively, whereas subjects with GDM and MODY weaned off insulin completely.

**Conclusion:**

The insulin requirements for pregnancy complicated with T1DM and MODY were higher than those for T2DM and GDM. In the subjects with PGDM, the insulin requirement and percentage of preprandial insulin increased gradually from early to mid- and late pregnancy.

## Introduction

Hyperglycemia in pregnancy (HIP) could be classified as pre-gestational diabetes (PGDM), gestation diabetes mellitus (GDM), and diabetes in pregnancy (DIP) (1). Insulin is commonly prescribed as the first-line medication when lifestyle modification alone failed to achieve glycemic targets. Women with HIP who had to treat with insulin showed worse adverse maternal and infant outcomes, including a smaller gestational age at delivery and a higher neonatal ICU admission rate (2, 3). However, few studies had evaluated daily insulin dosage, composition, and change in insulin dosage during pregnancy. We aimed to compare the differences of maternal and neonatal outcomes among women with different types of HIP. In addition, we explored total daily dosage and percentage change in preprandial and basal insulin during pregnancy and postpartum period.

## Materials and methods

### Subjects

The study population comprised women with HIP attending Peking Union Medical College Hospital (PUMCH), Beijing, China, from April 2019 to October 2021. Subjects with renal or liver disease, tumor, and polycystic ovarian syndrome (PCOS) and those taking medication known to affect glycemic metabolism were excluded. Comorbidity data included the presence of hypertension or hypothyroidism.

**Figure 1:**
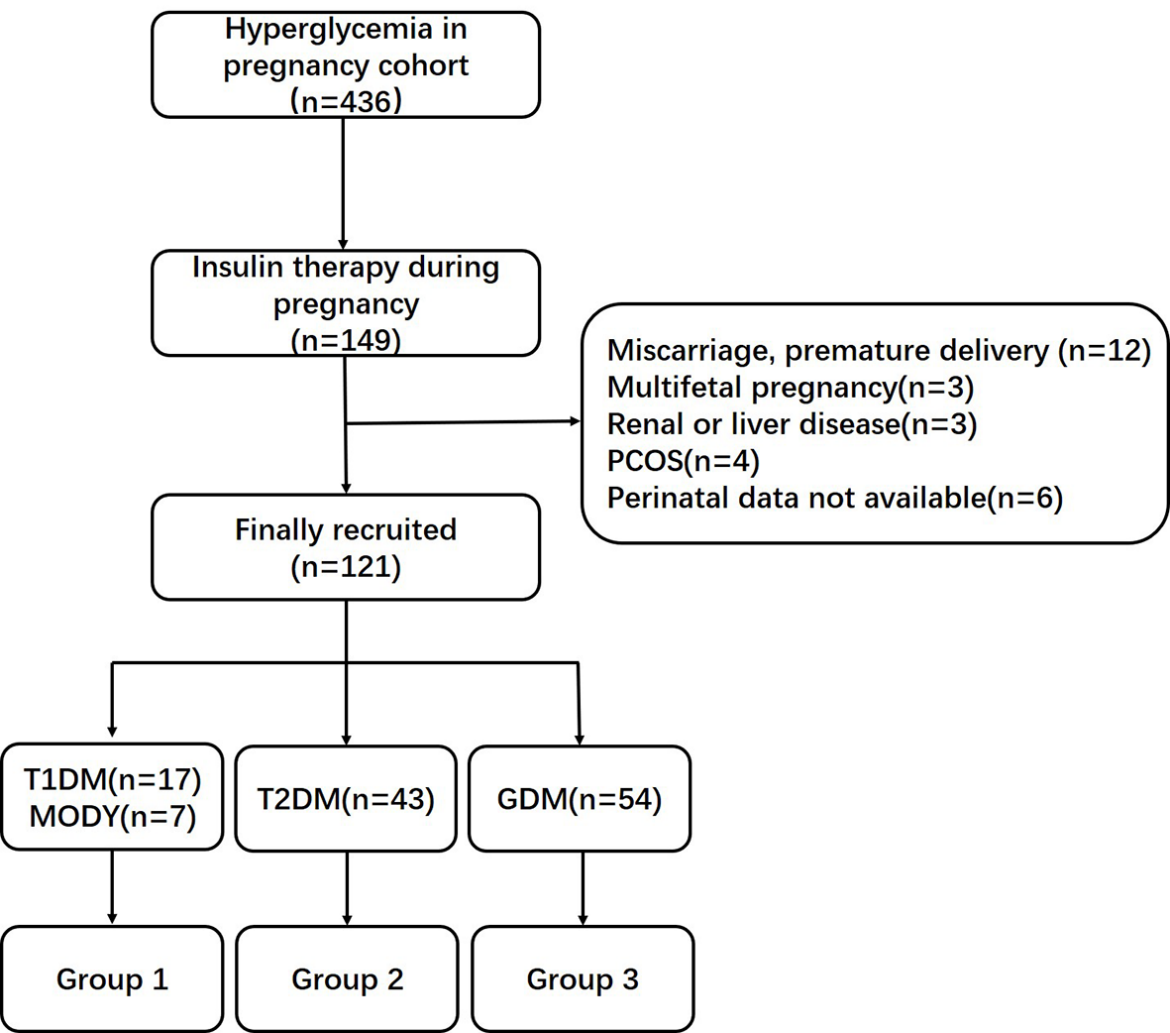
Flow chart of cohort selection. PCOS, polycystic ovarian syndrome; T1DM, type 1 diabetes mellitus; MODY, maturity onset diabetes of the young; T2DM, type 1 diabetes mellitus; GDM, gestational diabetes mellitus.

In total, 17 women with type 1 diabetes mellitus (T1DM), 43 women with type 2 diabetes mellitus (T2DM), 54 women with GDM, and seven women with a genetic diagnosis of maturity onset diabetes of the young (MODY, 3 GCK-MODY, 1 HNF1A-MODY, 1 PDX1-MODY, 1 HNF1B-MODY, and 1 KLF11-MODY) who had given birth to single live babies at term were recruited. The screening of MODY was based on a predictive model previously published by our center (4). Only 2.5% of subjects with HIP (two cases of T1DM and one case of T2DM) were managed on the insulin pumps therapy. The oral glucose tolerance test was undertaken in 24–28 weeks of gestation to screen for GDM. Among pregnant women with T2DM, 51.2% (22 cases) of the subjects were treated with lifestyle interventions, 41.9% (18 cases) need oral hypoglycemic agents, and only 9.3% of subjects (four cases) were treated with insulin before pregnancy. The clinical characteristics between T1DM and MODY individuals were similar, including maternal age, duration of diabetes, body mass index (BMI) levels, lipid profile, and insulin dependency during pregnancy (**Supplementary Table 1**). In addition, the sample size of MODY subjects in this study was relatively small. To make the conclusion more persuasive and scientific, these two types of diabetes were combined as Group 1. Pregnant women with T2DM and GDM were classified as Group 2 and Group 3. The flow chart of cohort selection is shown in **Figure 1**.

### Clinical and laboratory measurements

The prospective interventions were composed of lifestyle management, self-monitoring of blood glucose (SMBG), and insulin therapy. All women with HIP were treated with medical nutritional counseling based on the Dietary Reference Intakes (DRI) (5) and Chinese Recommendation for Pregnancy with Diabetes Mellitus (6). Calorie intake should be calculated on the basis of pre-pregnancy BMI and desirable weight gain as follows: 30–35 kcal/kg desirable body weight for women with normal weight and 25–30 kcal/kg desirable body weight for women with overweight/obesity. The energy supply percentages of carbohydrate, protein, and fat were 40%–60%, 15%–20%, and 20%–30% of total calories, respectively, including a minimum of 175 g of carbohydrate, a minimum of 71 g of protein, and 28 g of fiber. SMBG was performed five to eight times per day including fasting, 1 hour postprandial, 2 hours postprandial, and bedtime. Targets for glycemic control were as follows: fasting plasma glucose (FPG), 3.9–5.3 mmol/L; 1-hour value (1h-PG), 6.1-7.8 mmol/L; and 2-hour value (2h-PG), 5.6-6.7 mmol/L. All the women with diabetes were encouraged to breastfeed. The criteria for pharmacological interventions postpartum were based on the Chinese Diabetes Society guidelines for nonpregnant adults [FPG ≥ 7 mmol/L, 2h-PG ≥ 10 mmol/L, and glycosylated hemoglobin (HbA1c) ≥ 7%] (7). In view of this, insulin therapy was the only option for postpartum hyperglycemia. This study was approved by the Ethics Committee of PUMCH and conducted in accordance with the Declaration of Helsinki (Ethics Approval Number: JS-3000D).

Diabetes history and treatment details were obtained from medical records. Both preprandial and basal insulin requirements were recorded for the first trimester, second/third trimester, or postpartum period. Data including maternal demographics, comorbidity, family history of diabetes (first-degree relatives), obstetric history, gestational age at delivery, gestational weight gain (GWG), neonatal weight, and neonatal complications were collated. High-performance liquid chromatography was used for the measurement of HbA1c.

### Statistical analysis

All analyses were performed in IBM SPSS software (version 26.0, Chicago, IL, USA). Normal distribution of the data was evaluated with Kolmogorov–Smirnov test. Continuous variables were described as mean ± standard deviation if normally distributed and as median (interquartile range) if not. Independent sample T-Test, Mann–Whitney U-test, one-way ANOVA test, Kruskal–Wallis H-test, and Wilcoxon rank sum test were used as appropriate. The Chi-square statistic was utilized for categorical variables, and the results are described as frequencies (number of cases) and percentages. Levels of statistical significance were considered as P < 0.05.

## Results

### Anthropometric parameters and perinatal outcomes

Comparison of anthropometric features and perinatal outcomes among the three groups is presented in **Table 1**. Compared with Group 2 and Group 3, Group 1 had a younger maternal age and a lower pre-pregnancy BMI. There was no difference in mode of conception, comorbidity prevalence, family history of diabetes, and obstetric history among the three groups.

**Table 1:** Anthropometric data and perinatal outcomes of participants by subgroups.

	Group 1	Group 2	Group 3
Cases	24	43	54
Maternal age (years)	32.48 ± 3.25	36.02 ± 4.95^*^	35.41 ± 3.49^*^
Course (years)	6.5 (3.0, 10.0)	2.0 (1.0, 3.8)^*^	NA
Natural conception [n (%)]	22 (91.7)	35 (81.4)	42 (77.8)
Pre-pregnancy BMI (kg/m^2^)	22.00 ± 3.34	26.71 ± 3.85^*^	23.78 ± 4.65^#^
Delivery BMI (kg/m^2^)	26.49 ± 3.48	30.28 ± 3.88^*^	28.34 ± 4.86^#^
Obstetric history [n (%)]	9 (37.5)	22 (51.2)	28 (51.9)
Comorbidity prevalence [n (%)]	10 (41.7)	11 (25.6)	18 (33.3)
Family history of diabetes [n (%)]	8 (33.3)	23 (53.5)	25 (46.3)
Gestational age at delivery (weeks)	38.0 (38.0, 39.0)	38.0 (37.0, 39.0)	38.0 (38.0, 39.0)
GWG (kg)	11.84 ± 4.81	9.43 ± 4.50	11.94 ± 5.54
HbA1c in the first trimester (%)	6.93 ± 1.82	6.46 ± 1.11	5.37 ± 0.37^*#^
GA in the first trimester (%)	18.02 ± 3.98	14.01 ± 2.32^*^	13.56 ± 2.42^*^
HbA1c in the second/third trimester (%)	5.30 ± 0.76	5.53 ± 0.45	5.34 ± 0.56
GA in the second/third trimester (%)	14.78 ± 2.34	13.46 ± 1.29^*^	13.73 ± 1.53
Cesarean section [n (%)]	11 (45.8)	25 (58.1)	27 (50.0)
Neonatal weight (g)	3,427.19 ± 687.83	3,337.76 ± 486.96	3,420.21 ± 506.99
Macrosomia (%)	3 (12.5)	4 (9.3)	7 (13.0)
Other complications or congenital malformation [n (%)]	5 (20.8)	6 (14.0)	4 (7.4)
Insulin therapy after gestation [n (%)]	17 (70.8)	8 (18.6)^*^	0 (0)^*#^

BMI, body mass index; GWG, gestational weigh gain; HbA1c, Glycosylated hemoglobin; GA, Glycated albumin.

*p < 0.05 compared to Group 1, #p < 0.05 compared to Group 2.

There was no significant difference in gestational age at delivery, GWG, cesarean section, neonatal weight, macrosomia, 1-min Apgar score, and other complications or congenital malformation. In first the trimester, glycated albumin (GA) and HbA1c in Group 1 were higher than those in Group 2 and Group 3. In the second/third trimester of pregnancy, there was no difference in level of HbA1c; however, GA of Group 1 was still higher than that of the other two groups.

### Changes in insulin requirements throughout pregnancy

**Figure 2:**
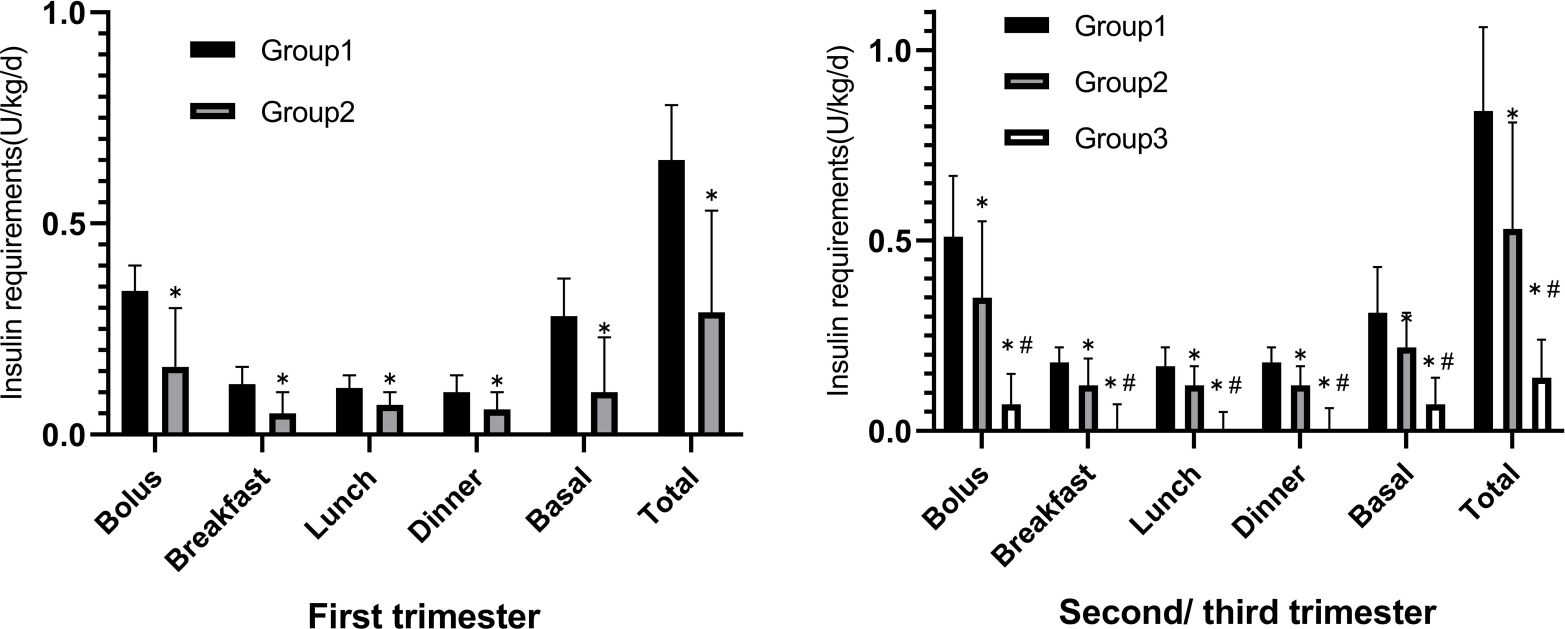
The comparison of daily Insulin dosage (U/kg/day) distribution of the three groups in the first trimester versus the second/third trimester. *p < 0.05 compared to Group 1, ^#^p < 0.05 compared to Group 2.

The comparisons of daily insulin requirements (U/kg/day) and dosage allocation of each group in the first trimester and the second/third trimester are shown in **Figure 2**. The preprandial, basal, and total insulin requirements of Group 1 [0.34 (0.21, 0.40) U/kg/day, 0.28 (0.16, 0.37) U/kg/day, and 0.65 (0.32, 0.78) U/kg/day] were higher than those of Group 2 [0.16 (0.00, 0.30) U/kg/day, 0.10 (0.00, 0.23) U/kg/day, and 0.29 (0.06, 0.53) U/kg/day] in the first trimester of pregnancy. The insulin requirements gradually increased in the second/third trimester and the dosages of Group 1 [0.51 (0.38, 0.67) U/kg/day, 0.31 (0.15, 0.43) U/kg/day, and 0.84 (0.56, 1.06) U/kg/day] remained higher than those of Group 2 [0.35 (0.20, 0.55) U/kg/day, 0.22 (0.09, 0.31) U/kg/day, and 0.53 (0.29, 0.81) U/kg/day] (**Supplementary Table 2**).

Because GDM screening was usually not performed until 24th gestational week, insulin therapy in Group 3 was usually initiated during the second/third trimester. In addition, the mealtime and basal insulin requirements [0.07 (0.00, 0.15) U/kg/day, 0.07 (0.05, 0.14) U/kg/day, and 0.14 (0.08, 0.24) U/kg/day] were significantly lower than those of Group 1 or Group 2.

All subjects with T1DM relied on insulin therapy postpartum, with postpartum total daily dose (TDD) reduced to 0.69 (0.48, 0.78) U/kg/day and an average reduction of 26.9% (19.0, 46.0). In contrast, only 18.6% of subjects with T2DM were insulin dependent after delivery, with TDD of 0.38 (0.27, 0.54) U/kg/day and an average reduction of 36.7% (26.9, 52.6) compared to prenatal requirements (**Supplementary Table 3**). All of the subjects with GDM and MODY weaned off insulin completely.

The proportion of daily insulin dosage was further compared among the three groups. It was found that preprandial insulin requirements of TDD were significantly higher in Group 2 [67.2% (51.8, 73.7)] and Group 1 [63.6% (54.9, 75.0)] when compared with that in Group 3 [50.0% (0.0, 66.7)] (P = 0.006) in the second/third trimester.

**Figure 3:**
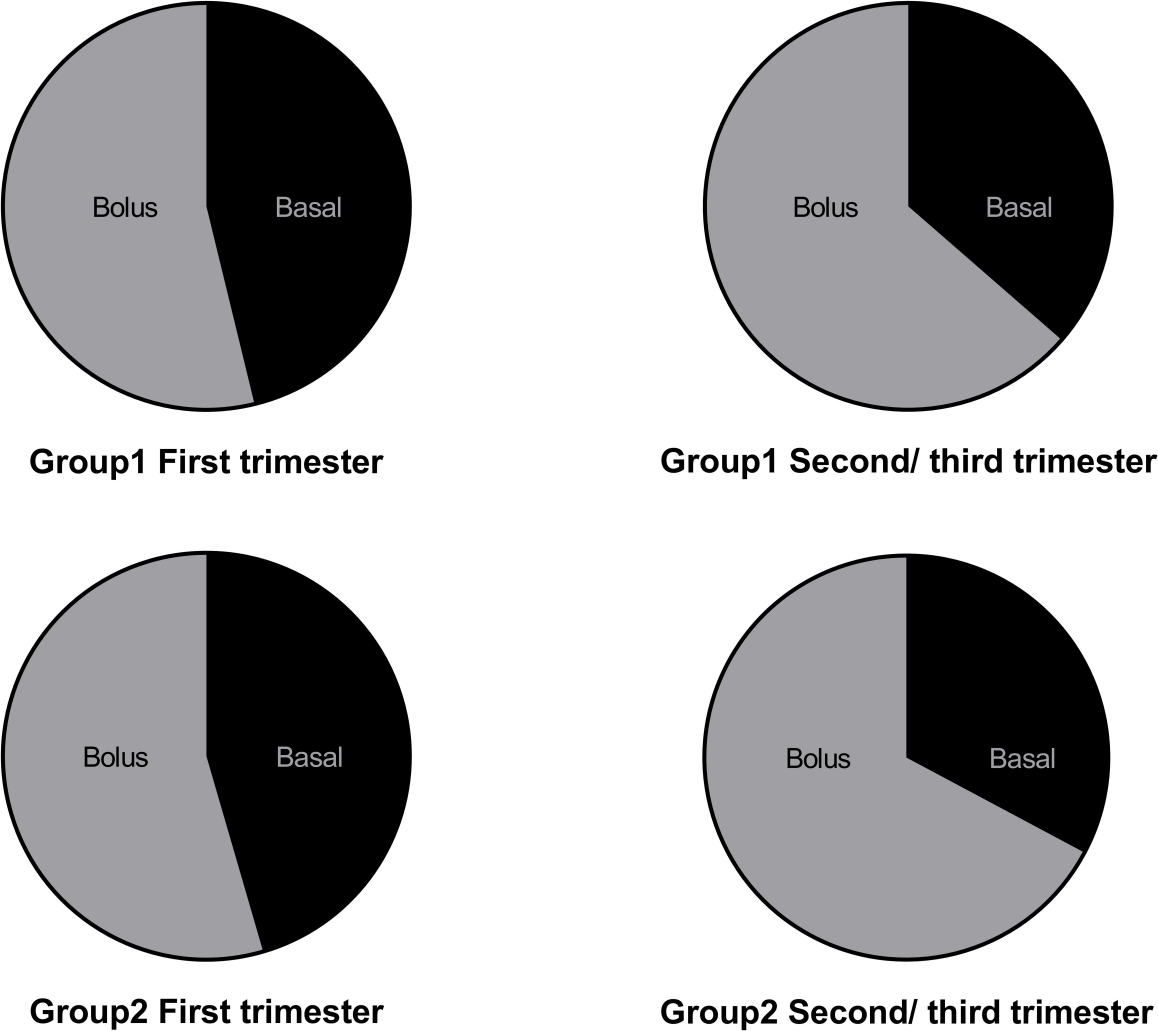
Comparison of percentage of preprandial insulin between Group 1 and Group 2.

There was no difference in preprandial insulin percentage between Group 1 and Group 2 in the first trimester of pregnancy. The percentage of preprandial insulin requirements increased from 53.8% (46.7, 60.0) and 54.5% (49.3, 62.9) of TDD in the first trimester to 63.6% (54.9, 75.0) and 67.2% (51.8, 73.7) of TDD in the second/third trimester, respectively (**Figure 3**).

## Discussion

HIP occurred worldwide and is closely related to the health problems in women and their offspring, such as maternal-infant complications and metabolic and cardiovascular diseases. Favorable glycemic control plays a major role in satisfactory perinatal outcomes.

Several risk factors for HIP have been identified, such as previous GDM, family history of diabetes, ethnicity, higher maternal age, pre-pregnancy obesity, or overweight. Excess neonatal and maternal short- and long- term complications are associated with HIP (8, 9). Furthermore, hyperglycemia is also correlated with inflammation including kisspeptin. Meanwhile, reduced kisspeptin level production may be a risk factor of GDM (10). Other studies have shown that kisspeptin may be used as a potential biomarker including GDM in maternal complications (11, 12). Concurrently assessing these risk factors may facilitate the identification of women at risk for HIP and the implementation of HIP prevention measures. In the future study, we will explore the effect of gestational biomarkers levels including kisspeptin on perinatal complications.

Lifestyle interventions such as dietary changes and physical activity were cornerstones in treating HIP. There were issues regarding efficacy and safety of oral pharmacotherapy during pregnancy; thus, insulin is commonly prescribed as the first-line treatment following lifestyle intervention. There are two insulin delivery systems for HIP: multiple daily injections (MDI) and continuous subcutaneous insulin infusion (CSII). According to recent studies, pregnant women with T1DM on CSII therapy have shown lower insulin requirements and better glycemic control compared with those on MDI (13, 14). Some studies have found no significant difference in perinatal outcomes between MDI and CSII groups (13, 15). Of note, at least one study suggested that MDI were superior to CSII in achieving lower HbA1c levels (16). A meta-analysis also reported that CSII therapy was associated with an increasing risk of higher GWG and large for gestational age (14).

Well-controlled HIP was usually defined as GA < 15.7% (17) or HbA1c < 6.0% (18). The present study showed that 73.8% of subjects achieved GA targets in the first trimester, whereas in the second/third trimester, the control rate increased to 88.2%. Likewise, the proportion of subjects with HbA1c lower than 6.0% increased from 54.4% to 90.2% (**Supplementary Table 2**). Overall, from the first trimester to the second/third trimester, the averaged decrease of GA and HbA1c was 0.40% and 0.50%, respectively. In this study, the incidence of macrosomia was 12.5%, 9.3%, and 13.0% in the three groups, respectively (P = 0.928), which were not higher than the incidence of macrosomia in well-controlled GDM from the same center previously reported (19). These results also showed that subjects with HIP may benefit from strict glycemic control.

Although there were no significant statistical differences, pregnant women with insulin-treated T2DM seemed to have a relatively lower incidence of macrosomia and less GWG. It has reported that maternal dysglycemia in early pregnancy associated with poorer pregnancy outcomes (9, 20, 21). Therefore, we speculated that early intervention on glycemic control and GWG had a greater effect on neonatal birth weight.

Insulin requirements during pregnancy and postpartum vary greatly for the alteration of insulin sensitivity, bodyweight, and appetite, so it is important to find out the change rules of dosage and composition. Our study found that, from the first trimester to the second/third trimester, the increasing daily dosage in Group 1 and Group 2 was 0.25 (0.15, 0.82) U/kg/day and 0.24 (0.06, 0.42) U/kg/day, respectively, with an increase in the total insulin dosage of 30.0% (14.6, 82.2) and 56.4% (5.1, 130.0); in addition, the increasing preprandial dosage in Group 1 and Group 2 was 0.21 (0.15, 0.29) U/kg/day and 0.18 (0.00, 0.29) U/kg/day, respectively, with an increase in the preprandial dosage of 64.3% (30.0, 107.9) and 50.9% (0, 122.1) (**Supplementary Table 3**).

Moreover, the percentage of preprandial insulin increased about 10% in both groups. In early stage of pregnancy, improved insulin sensitivity might be related to the decrease of food intake and depletion of glucose and glycogen reserves (22). In the second and third trimesters of pregnancy, decreased insulin sensitivities were promoted by the progressive increase in placental hormones, such as estrogen, progesterone, prolactin, and growth hormone (23). Serum lipid levels were elevated during the second/third trimester of pregnancy (24, 25). Both of them results in higher maternal post-prandial blood glucose levels (9). A relatively high amount of carbohydrates recommended by DRI may also play a role in high percentage of preprandial insulin requirements.

Insulin requirements were drastically reduced after delivery of the placenta. Our study found that insulin was completely withdrawn in all the subjects with MODY in Group 1, 81.4% with T2DM, and 100% with GDM. The daily dosage reduction in T1DM and T2DM was 0.21 (0.13, 0.44) U/kg/day and 0.31 (0.19, 0.44) U/kg/day, respectively, with a decline by 26.9% (19.0, 46.0) and 36.7% (26.9, 52.6). In addition, the decrease in preprandial dosage was 30.5% (19.4, 38.9) and 34.7% (14.5, 50.2) in T1DM and T2DM, respectively (**Supplementary Table 4**). Therefore, postpartum insulin dosage should be reduced substantially to avoid the risk of hypoglycemia.

This study does have some limitations. The number of enrolled subjects was relatively limited, and data about changes in insulin requirements in each gestational week were not recorded. A small number of pregnant women with miscarriage, multiple pregnancies, and incomplete records were excluded for this study, which may lead to bias in the results. In addition, the specific mechanism of increase in the percentage of preprandial insulin in the second/third trimester of pregnancy still required further research and remained to be explored.

## Conclusion

In summary, pregnancies complicated by T1DM and MODY had higher insulin requirements than that by T2DM and GDM. Percentage of preprandial insulin was roughly equivalent to basal insulin in the first trimester for PGDM but markedly increased in the second/third trimester. All women complicated by T1DM and approximately one in five women with T2DM continued to be treated with insulin administration after delivery, whereas women with GDM weaned off insulin completely. Therefore, postpartum insulin dosage should reduce timely to avoid hypoglycemia. Clarifying the changes in insulin dosing throughout pregnancy in women with HIP may contribute to achieve optimized glycemic control for physicians and patients.

## Data availability statement

The raw data supporting the conclusions of this article will be made available by the authors, without undue reservation.

## Ethics statement

This study was reviewed and approved by Ethics Committee of PUMCH. The patients/participants provided their written informed consent to participate in this study.

## Author contributions

FP carried on the study design, the data collection, the interpretation of the data, and the preparation of the manuscript. CR carried on the data collection and the analysis and interpretation of the data and drafted the manuscript. All authors contributed to the article and approved the submitted version.

## Funding

This research was funded by the CAMS Innovation Fund for Medical Science (CIFMS), grant number 2021-1-I2M-028 and 2021-I2M-C&T-B-010; Diabetes Mellitus Research Fund Program from Shanghai Medical and Health (SHMHDF), grant number DMRFP II_09.

## Acknowledgments

We would like to thank all pregnant women who participated in this PUMCH study.

## Conflict of interest

The authors declare that the research was conducted in the absence of any commercial or financial relationships that could be construed as a potential conflict of interest.

## Publisher’s note

All claims expressed in this article are solely those of the authors and do not necessarily represent those of their affiliated organizations, or those of the publisher, the editors and the reviewers. Any product that may be evaluated in this article, or claim that may be made by its manufacturer, is not guaranteed or endorsed by the publisher.
