# Supplementary material for: Comparison of insulin requirements across gestation in women with hyperglycemia in pregnancy: A prospective cohort study
Source: Front Endocrinol (Lausanne). 2022 Oct 20;13:1013663. doi: 10.3389/fendo.2022.1013663 (PMC9633005; doi:10.3389/fendo.2022.1013663)
Supplement: Supplementary file 1 [file Table_1.docx]

Supplementary Material

# Supplementary Tables

**Supplementary table 1 General characteristics of T1DM and MODY**

|  | T1DM | MODY | p |
| --- | --- | --- | --- |
| Cases | 17 | 7 |  |
| maternal age (years) | 31.88±3.16 | 33.57±3.26 | 0.251 |
| Pre-pregnancy BMI (kg/m2) | 22.71±3.05 | 20.22±3.59 | 0.098 |
| TC (mmol/L) | 4.10±1.01 | 4.15±0.49 | 0.928 |
| TG (mmol/L) | 0.75±0.39 | 0.95±0.42 | 0.307 |
| HDL-C (mmol/L) | 1.56±0.47 | 1.65±0.43 | 0.711 |
| LDL-C (mmol/L) | 2.13±0.75 | 1.98±0.86 | 0.738 |

T1DM, type 1 diabetes mellitus; MODY, maturity onset diabetes of the young; BMI, body mass index; TC, total cholesterol; TG, triglycerides; HDL-C, high-density lipoprotein cholesterol; LDL-C, low-density lipoprotein cholesterol

**Supplementary table 2 Changes and compliance rate of GA and HbA1c during pregnancy**

|  | Group1 | Group2 | Group3 | Total |
| --- | --- | --- | --- | --- |
| Compliance rate of GA in first trimester | 38.1% (8/21) | 83.8% (31/37) * | 88.5% (23/26) * | 73.8% (62/84) |
| Compliance rate of GA in second/third trimester | 73.3% (14/19) | 92.5% (37/40) | 90.7% (39/43) | 88.2% (90/102) |
| Absolute change in GA (%) | 1.90(0.95, 3.75) | 0.15(-0.80, 1.13) * | 0.30(-0.53, 0.90) * | 0.40(-0.50,1.70) |
| Percent change in GA (%) | 12.61(5.77,22.26) | 1.00(-6.27, 6.86) * | 2.21(-3.04, 6.35) * | 2.88(-3.49, 10.06) |
| Compliance rate in HbA1c in first trimester | 33.3% (7/21) | 48.3% (14/29) | 88.9%(16/18) *# | 54.4% (37/68) |
| Compliance rate in HbA1c in second/third trimester | 92.9% (13/14) | 88.2% (15/17) | 90.0% (27/30) | 90.2% (55/61) |
| Absolute change in HbA1c (%) | 0.80(0.10,1.15) | 0.50(-0.20, 1.30) | 0.05(-0.05,0.50) | 0.50(0.00, 0.95) |
| Percent change in HbA1c(%) | 12.12(1.52, 19.06) | 7.81(-3.51, 19.40) | 1.0(-0.96, 8.51) | 7.81(0.00, 16.72) |

GA, Glycated albumin; HbA1c, Glycosylated hemoglobin.

* p＜0.05 compared to Group1，# p＜0.05 compared to Group 2

**Supplementary table 3 Changes of insulin requirements of Group1 and Group2 from first to second/third trimester of pregnancy**

|  | Group1 | Group2 | p |
| --- | --- | --- | --- |
| First trimester |  |  |  |
| preprandial | 0.34(0.21, 0.40) | 0.16(0.00, 0.30) | 0.007 |
| Basal | 0.28(0.16, 0.37) | 0.10(0.00, 0.23) | 0.005 |
| Total | 0.65(0.32, 0.78) | 0.29(0.06, 0.53) | 0.000 |
| Second/third trimester |  |  |  |
| preprandial | 0.51(0.38, 0.67) | 0.35(0.20, 0.55) | 0.000 |
| Basal | 0.31(0.15, 0.43) | 0.22(0.09, 0.31) | 0.000 |
| Total | 0.84(0.56, 1.06) | 0.53(0.29, 0.81) | 0.000 |
| Absolute change of preprandial insulin (U/kg/d) | 0.21(0.15,0.29) | 0.18(0.00,0.29) | 0.245 |
| Percent change of preprandial insulin (%) | 64.3(30.0,107.9) | 50.9(0,122.1) | 0.907 |
| Absolute change of TDD (U/kg/d) | 0.25(0.15,0.82) | 0.24(0.06,0.42) | 0.917 |
| Percent change of TDD (%) | 30.0(14.6,82.2) | 56.4(5.1,130.0) | 0.370 |

TDD, total daily dose.

**Supplementary table 4 Changes of postpartum insulin requirements compared with second/third trimester of pregnancy**

|  | Group1 | Group2 | p |
| --- | --- | --- | --- |
| postpartum |  |  |  |
| preprandial | 0.38(0.26, 0.44) | 0.25(0.17,0.35) | 0.097 |
| Basal | 0.27(0.17, 0.32) | 0.11(0.10, 0.23) | 0.028 |
| Total | 0.69(0.48, 0.78) | 0.38(0.27, 0.54) | 0.049 |
| proportion of preprandial insulin | 58.2(54.6, 67.2) | 63.3(54.7, 72.9) | 0.366 |
| Percent change of proportion of preprandial insulin (%) | 3.7(-6.6, 8.6) | -2.2(-10.5, 3.5) | 0.265 |
| Absolute change of preprandial insulin (U/kg/d) | 0.15(0.07, 0.24) | 0.18(0.05, 0.22) | 0.868 |
| Percent change of preprandial insulin (%) | 30.5(19.4, 38.9) | 34.7(14.5, 50.2) | 0.815 |
| Absolute change of TDD (U/kg/d) | 0.21(0.13, 0.44) | 0.31(0.19, 0.44) | 0.443 |
| Percent change of TDD (%) | 26.9(19.0, 46.0) | 36.7(26.9, 52.6) | 0.224 |

TDD, total daily dose.
